# Novel Insights into Molecular Mechanisms of Chronic Pain

**DOI:** 10.3390/cells9102220

**Published:** 2020-10-01

**Authors:** Ellen Niederberger

**Affiliations:** Pharmazentrum Frankfurt/ZAFES, Institut für Klinische Pharmakologie, Klinikum der Goethe-Universität Frankfurt, Theodor Stern Kai 7, 60590 Frankfurt am Main, Germany; e.niederberger@em.uni-frankfurt.de; Tel.: +49-69-6301-7616; Fax: +49-69-6301-7636

**Keywords:** pain, inflammation, cancer, neuropathy

## Abstract

Pain is the most frequent cause triggering patients to visit a physician. The worldwide incidence of chronic pain is in the range of 20% of adults, and chronic pain conditions are frequently associated with several comorbidities and a drastic decrease in patients’ quality of life. Although several approved analgesics are available, such therapy is often not satisfying due to insufficient efficacy and/or severe side effects. Therefore, novel strategies for the development of safe and highly efficacious pain killers are urgently needed. To reach this goal, it is necessary to clarify the causes and signal transduction cascades underlying the onset and progression of the different types of chronic pain. The papers in this Special Issue cover a wide variety of mechanisms involved in different pain types such as inflammatory, neuropathic or cancer pain. Therefore, the results summarized here might contribute to a better understanding of the mechanisms in chronic pain and thereby to the development of novel therapeutic strategies for pain patients.

Pain may arise from different causes and is a symptom of many underlying diseases. The sensation of pain can be triggered by acute nociceptive stimulation, inflammation, nerve damage or cancer. Acute pain is characterized by an obvious stimulus and a short duration and is therefore an important warning and protection mechanism of the body. In contrast, pathophysiological pain which lasts for several weeks, months or years constitutes a social and ecological problem causing a drastic decrease in the quality of life for the patients and a high cost for the health systems. Although several analgesic drugs are available, treatment is often hampered by side effects or insufficient efficacy [1,2]. Therefore, it is necessary to investigate the detailed mechanism of different pain types and to develop novel treatment approaches. This Special Issue is composed of six original research studies and two reviews which cover a variety of different pain types. New aspects on the mechanisms and treatment of inflammatory nociception, neuropathic and tumor pain are provided. The studies summarized here can therefore increase our knowledge on the different, clinically relevant pain types and might put forward the development of novel treatment strategies for pain patients.

One study dealt with the principle action of an important nociceptor protein which is involved in acute and pathophysiological pain. In particular, the group of Sinica et al. intended to investigate differences in the characteristics of human and mouse orthologues of the transient receptor potential ankyrin 1 channel (TRPA1). This protein is stimulated by noxious heat and noxious cold as well as chemical stimulation, however, its temperature-sensitive properties appear to differ between species [3]. The authors used electrophysiological and modeling approaches to compare human and mouse TRPA1. They could confirm that human and mouse orthologues are activated by heat, suggesting that a heat-sensitive domain occurs in both human and mouse orthologues. However, kinetically distinct components of voltage-dependent gating are differentially modulated by heat and cold. Furthermore, mouse and human TRPA1 could be activated by cold after simultaneous application of voltage and heat. The results of this study indicate a conserved molecular mechanism for the temperature- and voltage-dependent gating of TRPA1 across different species which is conversely discussed in the published literature. The authors suggest that variability in TRPA1 temperature responsiveness is rather due to allosteric mechanisms [4].

The group of Gross et al. used models of inflammatory nociception in mice to investigate a potential function of the small GTPase Rab27a. Rab27a has been described for its expression in neurons and its contribution to the regulation of exocytosis in various cell types [5] but its role in pain had not been investigated before. Rab27a expression was detected in nociceptive neurons of the spinal cord and the dorsal root ganglia (DRGs) of naïve mice, thus supporting a potential role in pain. The authors applied different models of inflammatory nociception including injection of formalin, zymosan or CFA into the hind paw of mice, respectively. For further experiments, Rab27a wild type and knock-down mice were used to assess differences in nociceptive behavior. The Rab27a wild type and knock-down mice did not show differences in the neuronal composition and were also similar in their acute nociceptive behavior. In addition, rapid sensitization of pain pathways, as occurs after formalin injection, was unaltered in the Rab27a knock-out mice. In contrast, inflammatory mechanical hyperalgesia as well as spontaneous pain behavior induced by zymosan or CFA, respectively, was significantly reduced in Rab27 mutant mice. These data strongly point to a novel role of Rab27a in inflammatory nociception [6].

The studies of Cohnen et al. and Lucarini et al. both lie at the interphase between neuropathic pain and painful inflammatory mechanisms. The progression of cancer may lead to the damage of nerves by the tumor itself or by metastasis which grow into the proximity of nerves [7]. The underlying mechanisms for this tumor-induced neuropathic pain are not completely elucidated. It has been suggested several times that pain is due to a mechanical compression or invasion of tumor cells into the affected peripheral nerves. The group of Cohnen et al. investigated the effect of different tumor cell types implanted near the sciatic nerve on neuronal damage and the development of pain. Their results showed that melanoma, adenocarcinoma and fibrosarcoma cells are inducing sensory deficits as indicated by mechanical hypersensitivity and thermal hyposensitivity. These effects on pain sensitivity were independent of infiltration of cancer cells into the sciatic nerve. Changes in pain sensitivity are rather due to an increase in immune cells which then locate to the outside of the sciatic nerve and cause a local inflammatory reaction. In addition, after tumor cell implantation, microlesions in the epineurom of the sciatic nerve were observed associated with reduced levels of perineural tight junction proteins such as ZO-1 and claudin-1 and an increased uptake of fluorescence-labeled nanoparticles. A further lipidomic approach indicated the damage of neuronal myelin sheaths, decreased energy metabolism and the loss of epineural adipocytes which have an important protective role in peripheral nerves. The authors concluded that inhibition of inflammatory processes at the sciatic nerve or preserving peri- and epineural integrity might constitute suitable approaches to decrease tumor-induced pain [8]. 

Visceral pain is often a result of inflammatory bowel disease (IBD) or inflammatory bowel syndrome (IBS). However, pain intensity and the extent of inflammation in IBD or IBS do not correlate very well and current treatments are not sufficient [9]. The study of Lucarini et al. focused on the interplay of the bowel and the nervous system and potential treatment approaches in a rat model of painful colitis induced by injection of 2,4-dinitrobenzenesulfonic acid (DNBS). Using this model, the authors could show that abdominal pain is a mixed-type nociception consisting of inflammatory and neuropathy mechanisms in both the peripheral and central nervous systems. Histologically, they observed a partial restitution of the tunica mucosa, transmural collagen deposition, infiltration of mast cells and eosinophils, and upregulation of substance P (SP)-positive nerve fibers, which were surrounded by immune cells. In the spinal cord, a significant activation of microglia and astrocytes was determined. From their results, the authors concluded that persistence of abdominal pain in a colitis model is due to maladaptive plasticity of the enteric, peripheral and central nervous systems [10].

Most of the published papers in this Special Issue focused on mechanisms of neuropathic pain. Neuropathic pain is often associated with dysfunctional reinnervation after peripheral nerve injury [11]. Two studies assessed reinnervation processes with one study focusing on the mode of sprouting of DRG neurons and the other one on a potential treatment approach with repurposed drugs which enhance neuronal regeneration. The group of Leibovich et al. adapted a multispectral retrograde labeling technology [12] to label DRGs (“Pain Bow”) which then allowed the characterization of the amount and type (nociceptive, non-nociceptive) of DRG neurons innervating specific areas in the rat’s plantar hind paw skin. Using this technique, they assessed the reinnervation patterns in the rat paw after applying the SNI model of neuropathic pain. Their results revealed that mainly large non-nociceptive neurons of dorsal root ganglia contribute to the reinnervation of the affected areas during the development of hyperalgesia and allodynia. In addition, they could show a decrease in the number of DRG neurons that innervate these areas. These changes in innervation could be correlated with symptoms of neuropathic pain. In a further approach, they enhanced the sprouting of nerves by the drug oubain and observed that this treatment drastically reduced the neuropathic pain behavior. From these results, it can be concluded that dysfunctional reinnervation of injured areas is involved in the development of neuropathic pain and that pharmacological support of reinnervation, e.g., by oubain, might exert a useful approach for the treatment of nerve injury-induced pain [13]. David Romeo-Guitart and Caty Casas described a new approach to modulate axonal regeneration and neuropathic pain at the same time by the use of a drug combination called NeuroHeal which has been identified using machine learning [14]. NeuroHeal is composed of two repurposed drugs, Acomprostate and Ribavirin. In models of spared nerve injury and nerve crush in rats, NeuroHeal alleviated neuropathic pain behavior and enhanced sensory axon regeneration. As molecular mechanisms of the drug effects, the authors suggest a resolution of cell-protective autophagy, a modulation of P2X4-BDNF-KCC2 pathways and a decrease in glia activation. These effects contribute to restoration of axonal integrity, on the one hand, and inhibition of neuropathic pain, on the other. The combination of the drugs showed superior effects compared to administration of either drug alone [15].

In addition to the original research articles, there are two review articles which focus on mechanisms of neuropathy. Asiri et al. summarized information on the role of amyloid proteins which contribute to painful peripheral neuropathy by the formation of tissue deposits. Aggregation of amyloid fibril-forming proteins affects cells involved in amyloid protein production or clearing, vascular cells and Schwann cells and thereby induces tissue dysregulation and damage, e.g., in the nervous system. This disruption of the homeostasis of diverse cell types together with the neurotoxic action of amyloids contributes to the damage of neurons. Therefore, aggregated amyloids have been associated with amyloid neuropathies [16]. Several diseases based on acquired (e.g., in type II diabetes) or familial (e.g., familial amyloid polyneuropathy (FAP)) extracellular deposits of aggregated, misfolded proteins are known and occasionally show neuropathic pain as one of the symptoms [17,18]. As potential mechanisms, the authors discuss protein–membrane interactions, ER stress, mitochondrial dysfunction and inflammatory mechanisms in particular with contribution of macrophages and microglia, and disturbances in autophagic processes as well as microangiopathy. With regard to the collected data, the authors suggest that amyloid disturbances might be involved in neuropathy in some common diseases which have not yet been considered as amyloid neuropathies and that further research in this field is very important [19]. 

The group of Cao et al. reviewed studies dealing with the role of brain derived neurotrophic factor (BDNF) and its receptors in neuropathic pain. BDNF is an important neurotrophin in the nervous system contributing to developmental processes as well as neuronal survival and functional recovery after nerve damage. Furthermore, it has been associated with pain processing in the CNS several times [20]. Tyrosine receptor kinase B (TrkB) is a major receptor for BDNF and is expressed in three different isoforms (a full length TrkB.FL, and two truncated forms, TrkB.T1 and TrkB.T2). TrkB.FL and TrkB.T2 are mainly expressed in neurons, and TrkB.T1 shows a high expression in astrocytes. While BDNF-induced activation of the full length receptor TrkB.FL has neuroprotective effects and promotes cell survival after nerve injury, mutations in TrkB.FL are associated with neurodegenerative diseases [21]. TrkB.T1 and TrkB.FL can form heterodimers in which TrkB.T1 acts as an inhibitor of TrkB.FL due to its lack of kinase activity. Consequently, upregulation of TrkB.T1 in response to injury and inflammation may contribute to harmful effects and has been associated with neuropathic pain. In contrast, TrkB.T1 inhibition is linked to reduced neuropathic pain. [22]. As one potential mechanism for the TrkB.T1 effects, the authors suggest its nerve injury-induced upregulation in astrocytes where it modulates calcium release and interacts with neurotrophins, thereby influencing the cell cycle. This might subsequently cause neuronal cell death, immune cell proliferation/activation and inhibition of cell regeneration and repair. The authors conclude that a complete deletion of TrkB receptors as a therapeutic measure in pain might be harmful due to the protective effects of TrkB.FL. However, a specific deletion of the TrkB.T1 receptor could be associated with beneficial effects such as improved functional recovery and a decrease in pain after nerve injury. Therefore, they suggest that this truncated receptor might constitute a suitable therapeutic target for several neurological diseases and neuropathic pain [23]. 

In conclusion, this Special Issue once more provides evidence that “pain” is a multifactorial disease, in which molecular mechanisms are still far from being completely elucidated. The articles add new knowledge as well as potential starting points for further studies which will then further put forward the understanding of pain signaling as well as the development of novel treatment strategies for pain patients.
